# Ambient air pollution exposure linked to long COVID among young adults: a nested survey in a population-based cohort in Sweden

**DOI:** 10.1016/j.lanepe.2023.100608

**Published:** 2023-03-07

**Authors:** Zhebin Yu, Sandra Ekström, Tom Bellander, Petter Ljungman, Göran Pershagen, Kristina Eneroth, Inger Kull, Anna Bergström, Antonios Georgelis, Massimo Stafoggia, Olena Gruzieva, Erik Melén, the BAMSE COVID-19 Study Group, Catarina Almqvist, Niklas Andersson, Natalia Ballardini, Anna Bergström, Sophia Björkander, Petter Brodin, Anna Castel, Sandra Ekström, Antonios Georgelis, Lennart Hammarström, Qiang Pan-Hammarström, Jenny Hallberg, Christer Jansson, Maura Kere, Inger Kull, André Lauber, Alexandra Lövquist, Erik Melén, Jenny Mjösberg, Ida Mogensen, Lena Palmberg, Göran Pershagen, Niclas Roxhed, Jochen Schwenk

**Affiliations:** a Institute of Environmental Medicine, Karolinska Institutet, Stockholm, Sweden; b Centre for Occupational and Environmental Medicine, Region Stockholm, Stockholm, Sweden; c Department of Cardiology, Danderyd Hospital, Stockholm, Sweden; d SLB-Analys, Environment and Health Administration, Stockholm, Sweden; e Department of Clinical Sciences and Education, Södersjukhuset, Karolinska Institutet, Stockholm, Sweden; f Sachs’ Children and Youth Hospital, Södersjukhuset, Stockholm, Sweden; g Department of Epidemiology, Lazio Regional Health Service, Rome, Italy

**Keywords:** Long COVID, Air pollution, Young adults, PM_2.5_

## Abstract

**Background:**

Post COVID-19 conditions, also known as long COVID, are of public health concern, but little is known about their underlying risk factors. We aimed to investigate associations of air pollution exposure with long COVID among Swedish young adults.

**Methods:**

We used data from the BAMSE (Children, Allergy, Environment, Stockholm, Epidemiology [in Swedish]) cohort. From October 2021 to February 2022 participants answered a web-questionnaire focusing on persistent symptoms following acute SARS-CoV-2 infection. Long COVID was defined as symptoms after confirmed infection with SARS-CoV-2 lasting for two months or longer. Ambient air pollution levels (particulate matter ≤2.5 μm [PM_2.5_], ≤10 μm [PM_10_], black carbon [BC] and nitrogen oxides [NO_x_]) at individual-level addresses were estimated using dispersion modelling.

**Findings:**

A total of 753 participants with SARS-CoV-2 infection were included of whom 116 (15.4%) reported having long COVID. The most common symptoms were altered smell/taste (n = 80, 10.6%), dyspnea (n = 36, 4.8%) and fatigue (n = 34, 4.5%). Median annual PM_2.5_ exposure in 2019 (pre-pandemic) was 6.39 (interquartile range [IQR] 6.06–6.71) μg/m^3^. Adjusted Odds Ratios (95% confidence intervals) of PM_2.5_ per IQR increase were 1.28 (1.02–1.60) for long COVID, 1.65 (1.09–2.50) for dyspnea symptoms and 1.29 (0.97–1.70) for altered smell/taste. Positive associations were found for the other air pollutants and remained consistent across sensitivity analyses. Associations tended to be stronger among participants with asthma, and those having had COVID during 2020 (versus 2021).

**Interpretation:**

Ambient long-term PM_2.5_ exposure may affect the risk of long COVID in young adults, supporting efforts for continuously improving air quality.

**Funding:**

The study received funding from the 10.13039/501100004359Swedish Research Council (grant no. 2020-01886, 2022-06340), the 10.13039/501100004359Swedish Research Council for Health, Working life and Welfare (FORTE grant no. 2017-01146), the 10.13039/501100003793Swedish Heart-Lung Foundation, 10.13039/501100004047Karolinska Institute (no. 2022-01807) and Region Stockholm (ALF project for cohort and database maintenance).

**Research in context**

**Evidence before this study**

Ambient air pollution exposure has been shown to increase the risk of corona virus disease (COVID-19) severity and mortality, but little is known about its association with post COVID-19 conditions (also known as long COVID). We searched PubMed and Web of Science with the combination of search terms: (‘air pollution’ OR ‘particulate matter’ OR ‘air pollutants’) AND (‘post COVID-19 conditions’ OR ‘long COVID’ OR ‘long-hauler’ OR ‘post COVID-19 syndrome’ OR ‘persistent symptoms’ OR ‘Post-Acute Sequelae of SARS-CoV-2 Infection’) up to September 30, 2022. Only one study from Brazil reported positive associations between long-term PM_2.5_ exposure and persistent symptoms after hospitalization for COVID-19 among 749 adult patients. No study has investigated such association among young adults with mild symptoms during acute infection.

**Added value of this study**

In a population-based prospective birth cohort, we assessed individual residence level both early-life and more recent exposure to air pollution (PM_2.5_, PM_10_, Black carbon and NOx) before the pandemic on the risk of having long COVID. We found that annual PM_2.5_ exposure in 2019 was significantly associated with increased risk of long COVID. The associations of PM_2.5_ tended to be stronger among participants with asthma and those having had COVID during 2020 (versus 2021).

**Implications of all the available evidence**

Our study provides evidence that air pollution is associated with the risk of having long COVID, suggesting broad adverse effects of ambient air pollution exposure and supporting efforts for continuously improving air quality.

## Introduction

As of September 2022, the COVID-19 pandemic has caused more than 612 million confirmed cases and resulted in more than 6.5 million deaths worldwide.^1^ Concerns have been raised about persistent symptoms following infection to SARS-CoV-2,^2^, ^3^, ^4^, ^5^, ^6^ typically fatigue, chronic cough, altered smell and taste which last for several months (also known as long COVID). These long-term sequelae of COVID-19 have been described as the next public health disaster.^7^ Although young adults are considered as having lower risk of hospitalization as well as low mortality rates due to COVID-19 compared with older adults,^8^ a recent meta-analysis including 22 studies found that the prevalence of persistent symptoms in post-COVID young people ranged from 15% to 47%^9^ regardless of the severity of the acute infection. There is an urgent need to identify potential modifiable risk factors for long COVID to help reduce the disease burden.

Ambient air pollution has been shown to be associated with spread of the corona virus,^10^, ^11^, ^12^ increased risk of COVID-19 incidence, disease severity and case fatality in ecological studies^13^, ^14^, ^15^, ^16^ and further confirmed by studies using individual-level data.^17^, ^18^, ^19^, ^20^, ^21^, ^22^, ^23^ Long-term exposure to air pollution may perturb immune response,^24^ increase autoantibody^25^ and induce microbiome imbalance,^26^ which are hypothesized mechanisms of long COVID pathogenesis.^27^ Beyond the direct effect, air pollution may also increase the risk of comorbidities^28^ linked to long COVID. Therefore, air pollution may directly or indirectly affect the risk of long COVID. However, little is known regarding the association between long-term air pollution exposure and the development of long COVID, and the epidemiological evidence remains scarce. To date, only one study from Brazil found that exposure to ambient particulate matter with diameter ≤2.5 μm (PM_2.5_) was associated with higher risk of dyspnea and fatigue among 749 hospitalized adult patients,^29^ but whether these associations also exist among non-hospitalized patients, especially young adults, remains unknown. Moreover, little is known about the importance of the timing of air pollution exposure, for example early-life exposure when the lungs are thought to be more susceptible to environmental exposure, or exposure later in life.^30^^,^^31^

Therefore, we examined the association between long-term exposure to air pollution estimated at the individual residence level in different exposure time-windows and development of long COVID in a population-based cohort of young adults in Sweden. In addition, we evaluated potential factors that may modify the association of air pollution with long COVID.

## Methods

### Study population

We used data from the BAMSE (Children, Allergy, Milieu, Stockholm, Epidemiology [in Swedish]) population-based prospective cohort,^32^ which recruited 4089 children born in 1994–1996 who have been subsequently followed with repeated questionnaires and clinical examinations. The 24-year follow-up^33^ was conducted during 2016–2019, with a total of 2270 participants attending both questionnaire survey and clinical examinations. These participants were further invited to the COVID-19 follow-up,^34^ which includes a web-questionnaire from August to November 2020 (phase 1), a clinical examination and web questionnaire from October 2020 to June 2021 (phase 2), and a new web questionnaire focusing on long COVID (response rate 68.7%, characteristics between respondents and non-respondents shown in Table S1) from October 2021 to February 2022 (phase 3) (Fig. S1).

The current study was conducted among the 2049 participants, who answered the phase 3 questionnaire of the COVID-19 follow-up, and included those 753 who had infection to SARS-CoV-2 according to either one of the following criteria: (1) positive results for SARS-CoV-2 polymerase chain reaction (PCR) testing up to the date of the questionnaire identified through data linkage of unique personal identifier codes to the SmiNet,^35^ which is the national registry of infectious diseases in Sweden; (2) positive results of anti-SARS-CoV-2 antibodies in the serum samples collected in phase 2^36^; (3) self-reported positive results of PCR, antibody or antigen test in phase 3. This study was approved by the Swedish Ethical Review Authority (approval 2020-0922) and all participants gave written informed consent. This study adhered to the Strengthening the Reporting of Observational Studies in Epidemiology (STROBE) reporting guideline with a completed checklist for observational studies in epidemiology.

### Outcomes

Based on the definition from the World Health Organization,^37^ in the current study long COVID was defined as self-report of any of the following symptoms after infection with SARS-CoV-2 lasting for two months or longer: shortness of breath or difficulty breathing, extreme fatigue, fever or feeling feverish, altered sense of smell and taste, headache, high resting heart rate or palpitations, cognitive impairment, gastrointestinal problems, muscle weakness, neurological symptoms, mental illness, pain and sleep disorders.

### Air pollution exposure assessment

Long-term exposure to PM_2.5_, particulate matter with diameter ≤10 μm (PM_10_), black carbon (BC), nitrogen dioxide (NO_2_) and nitrogen oxides (NO_x_) at the individual address level was estimated using a Gaussian air quality dispersion model and a wind model, both part of the Airviro air quality management system (https://www.airviro.com/airviro/). The Gaussian model calculates the horizontal distribution of air pollution concentrations 2 m above ground level. In densely populated areas, the calculations represent concentrations 2 m above roof level. The calculations use a variable grid size, between 35 × 35 m and 500 × 500 m, with the smallest grid size in the urban areas. In addition, a street canyon contribution was calculated for addresses located on busy inner-city streets flanked by contiguous high buildings using the Airviro street canyon model (until the year 2012; https://www.airviro.com/airviro/modules/) and the Operational Street Pollution Model, OSPM (from 2013 onwards; www.au.dk/OSPM). Meteorological data (climatological wind and temperature profiles) for the wind model were taken from a 50-m mast in southern Stockholm. As input to the dispersion modelling, emission inventories for the years 1990, 1995, 2000, 2011, 2015 and 2020 were used. For years in between, linear interpolation was used. The emission inventories include local emissions from road traffic (exhaust and non-exhaust), residential wood combustion, energy production, industrial processes, and other sources (eg, off-road machinery and agriculture) in Stockholm and Uppsala counties, described in detail elsewhere.^38^ In addition, annual average long-range contributions based on continuous measurements at regional background station were added to the locally modelled concentrations. More details of the dispersion modelling are provided in the eMethods of the Supplement.

Four pre-pandemic exposure time-windows were calculated: the 2019 annual average (i.e., immediately preceding the pandemic), the time-weighted average from the 16-year follow-up to the 24-year follow-up (calendar years covering 2010–2019), the time-weighted average from the 1-year to the 16-year follow-up (calendar years covering 1994–2013) and during the first year of life. The exposure estimation took into consideration time spent in different locations (home, day-care and school) up to 16 years of age as well as residential history during the time window of interest. NO_2_ was not used in the association analysis due to the high correlation with NOx, e.g., r = 0.99 in annual exposure 2019.

### Covariates

Demographic, clinical and COVID related information was derived from baseline questionnaire (date of birth, sex, municipality at birth), 24-year follow-up questionnaire (education level, occupation, active smoking, physical activity, general stress) and COVID-19 follow-up questionnaire (being bedridden or hospitalized due to COVID-19, anxiety during the pandemic). Body mass index (BMI) was calculated using the height and weight measured at the 24-year clinical examination. Overweight was defined as BMI ≥25 kg/m^2^. Physical activity levels were defined according to international Physical Activity Questionnaire (IPAQ). Allergic sensitization was based on IgE-levels against common food and/or airborne allergens in blood^39^ at the 24-year follow-up (≥0.35 kU/L defined as positive). Asthma was defined based on at least two of the three following criteria in the last 12 months^40^: (1) symptoms of wheezing (2) ever having a doctor's diagnosis of asthma (3) asthma medicine used occasionally or regularly. Individuals fulfilling the asthma definition at any follow-up to 24 years were classified as having asthma. Respiratory disorders before the pandemic were based on the questionnaire in the 24-year follow-up. Information regarding COVID-19 vaccination was derived through data linkage to the Swedish national vaccination register database. General stress was defined based on the perceived stress scale (PSS-10) consisting of 10 questions on how the participants perceived and handled stress or stressful situations in the last month^41^ at the 24-year follow-up. Anxiety due to COVID-19 was determined based on the answer to the question: ‘Have you felt increased anxiety due to COVID-19?’^41^

### Statistical analysis

Data were presented as frequency (percentage) for categorical variables and median (interquartile range) for continuous variables. Correlations between different air pollutants at different exposure windows were estimated using the Spearman correlation index. We used logistic regression models to estimate the association between long-term air pollution exposure and the presence of long COVID. Two models were determined *a priori*: Model 1 adjusted for age, sex and municipality at birth, and Model 2 further adjusted for education level, occupation, active smoking, overweight and physical activity at the 24-year follow-up. air pollution concentrations were entered into models as linear terms in the main analysis with Odds Ratios (ORs) and 95% confidence intervals (CIs) presented as per interquartile range (IQR) change. We also applied natural splines with three to six degrees of freedom to explore the exposure-response curves. Missing values for covariates were coded as a ‘missing’ category and included in the regression analysis to avoid loss of sample size (0.4% for education and 15.8% for physical activity).

Subgroup analyses were conducted to test whether the associations differed by sex, overweight, asthma status, allergic sensitization, respiratory disorder before the pandemic, COVID-19 disease severity (bedridden or not), calendar year of the COVID-19 disease (2020 or 2021). Interaction was tested by adding exposure-modifier interaction terms into the model. For sensitivity analyses, we: (1) excluded participants who self-reported having COVID-19 but without confirmation from the SmiNet or antibody data (n = 184); (2) additionally adjusted for short-term air pollution exposure before the date of PCR test sampling, using daily average concentration measured at urban background station located in central Stockholm; (3) additionally adjusted for area-level socioeconomic status data (using area-based median income from Statistics Sweden at the time of birth and in 2014, which is the most recent available year of the data); (4) additionally adjusted for general stress at 24-year follow-up; (5) additionally adjusted for anxiety during the pandemic; (6) additionally adjusted for COVID-19 reinfections; (7) using a wider definition of COVID-19 (not restricted to self-report positive test but also including self-report of ever having had COVID), thus increasing the sample size to 1076. (8) additionally adjusted for blood pressure at the 24-year follow-up. We also ran separate analyses for each symptom (only the three most frequent symptoms due to the sample size) as well as classified the symptoms into three symptom clusters (https://www.cdc.gov/coronavirus/2019-ncov/long-term-effects/index.html): general symptoms, respiratory and heart problems and neurological symptoms. Due to the relatively high correlations between air pollutants, ridge regression was used as the multi-exposure model to disentangle the potential independent effect of each air pollutant. All analyses were performed using R software, version 4.0.5, with two-sided p-values less than 0.05 indicating statistical significance.

### Role of the funding source

The sponsor of the study had no role in study design, data collection, data analysis, data interpretation, or writing of the report. The corresponding author had full access to all data in the study and had final responsibility for the decision to submit for publication.

## Results

A total of 753 participants were included in the current analysis, with median age of 26.5 years old and 457 (60.7%) were female. 359 participants had positive PCR results identified from SmiNet, 294 participants had positive antibody results while 658 participants self-reported having positive PCR, antibody or antigen test results (overlap in Venn diagram presented in Fig. S2). Basic characteristics of the participants are presented in Table 1. As many as 388 (51.5%) participants reported bedbound after infection with the SARS-CoV-2 virus, while only 26 (3.5%) participants had received vaccine before their infection. A total of 116 participants were classified as having long COVID with the highest frequency of symptom as altered sense of smell and taste (n = 80) followed by shortness of breath (n = 36) and extreme fatigue (n = 34).

**Table 1:** Characteristics of the study population with SARS-CoV-2 infection in the BAMSE cohort from August 2020 to February 2022 (N = 753).

Characteristics	Participants without long COVID (N = 637)	Participants with long COVID (N = 116)
Age^a^, median (IQR), years	26.5 (1.4)	26.5 (1.3)
Sex		
Male	255 (40.0)	41 (35.3)
Female	382 (60.0)	75 (64.7)
Education level at 24 years		
Elementary school or High school	233 (36.6)	46 (39.7)
University or college	401 (63.0)	70 (60.3)
Missing	3 (0.5)	0
Occupation at 24 years		
Student	360 (56.5)	61 (52.6)
Employed	237 (37.2)	51 (44.0)
Other	40 (6.3)	4 (3.4)
BMI at 24 years, median (IQR), kg/m^2^	22.5 (4.0)	22.1 (3.5)
Overweight (BMI ≥ 25 kg/m^2^) at 24 years	118 (18.5)	21 (18.1)
Current smoking at 24 years	137 (21.5)	28 (24.1)
Ever asthma up to 24 years	195 (30.6)	40 (34.5)
Having allergic sensitization at 24 years	229 (35.9)	46 (39.7)
General stress^b^ at 24 years, median (IQR)	14 (10)	15 (11)
Physical activity level^c^ at 24 years		
Low	78 (12.2)	11 (9.5)
Moderate	142 (22.3)	29 (25.0)
High	315 (49.5)	59 (50.9)
Missing	102 (16.0)	17 (14.7)
Bedridden due to COVID-19	309 (48.5)	79 (68.1)
COVID-19 re-infection	95 (14.9%)	33 (28.4%)
Anxiety during the pandemic	227 (35.6)	40 (34.5)
Had vaccine before infection to SARS-CoV-2	25 (3.9)	1 (0.9)
Symptoms of long COVID		
Any symptoms below	–	116 (100)
Shortness of breath or difficulty breathing	–	36 (31.0)
Extreme fatigue (physical or mental)	–	34 (29.3)
Altered sense of smell and taste	–	80 (69.0)
Headache	–	18 (15.5)
High resting heart rate or palpitations	–	16 (13.8)
Cognitive impairment/memory and concentration difficulties	–	19 (16.4)
Gastrointestinal problems	–	13 (11.2)
Muscle weakness	–	17 (14.7)
Neurological symptoms	–	5 (4.3)
Mental illness (depression, anxiety, feeling down)	–	24 (20.7)
Pain (chest, muscle, and joint pain)	–	19 (16.4)
Sleep disorders	–	17 (14.7)

Abbreviations: BMI, body mass index; IQR, interquartile range.

Data are presented as number (percentage) of participants unless otherwise stated.

a Age at the time of filling the phase 3 questionnaire.

b General stress was measured using the Perceived Stress Scale with a range of 0–40 points, higher points indicating higher level of stress.

c Physical activity levels were defined according to the International Physical Activity Questionnaire (IPAQ) at the time of 24-year follow-up. High-level defined as ≥7 h per week of moderate to vigorous activity or ≥3.5 h per week of vigorous activity; Moderate defined as ≥2.5 h per week of moderate to vigorous activity; Low defined as <2.5 h per week of moderate to vigorous activity.

Distribution of the air pollution exposure in three different exposure windows is presented in Table 2 and Table S2. The median level of air pollution concentrations was generally lower for the recent exposure (for PM_2.5_, 6.39 (IQR: 6.06–6.71) μg/m^3^; for PM_10_, 11.54 (IQR: 10.63–12.36) μg/m^3^; for BC, 0.34 (IQR: 0.30–0.37) μg/m^3^; for NO_2_, 9.01 (IQR: 6.50–11.69) μg/m^3^; for NO_x_, 10.47 (IQR: 7.39–14.27) μg/m^3^ of the 2019 annual average exposure), than for the exposure in the first year of life (for PM_2.5_, 8.89 (IQR: 8.37–9.57) μg/m^3^; for PM_10_, 14.65 (IQR: 13.36–16.38) μg/m^3^; for BC, 1.03 (IQR: 0.76–1.36) μg/m^3^; for NO_x_, 31.11 (IQR: 20.38–44.05) μg/m^3^). Concurrent air pollution estimates were highly correlated with each other while positively moderately correlated with air pollution estimates across other time windows (Fig. S3).

**Table 2:** Distribution of air pollution exposure for the participants in different time windows.

Exposure time window	Air pollutants	Median (25th percentile–75th percentile)
2019 annual average	PM_2.5_, μg/m^3^	6.39 (6.06–6.71)
	PM_10_, μg/m^3^	11.54 (10.63–12.36)
	BC, μg/m^3^	0.34 (0.30–0.37)
	NO_2_, μg/m^3^	9.01 (6.50–11.69)
	NO_x_, μg/m^3^	10.47 (7.39–14.27)
16-y to 24-y time weighted average^a^	PM_2.5_, μg/m^3^	5.49 (5.12–5.77)
	PM_10_, μg/m^3^	12.78 (11.80–13.60)
	BC, μg/m^3^	0.47 (0.47–0.55)
	NO_x_, μg/m^3^	12.20 (8.33–17.86)
1-y to 16-y time weighted average^a^	PM_2.5_, μg/m^3^	7.77 (7.30–8.18)
	PM_10_, μg/m^3^	13.22 (11.99–14.32)
	BC, μg/m^3^	0.80 (0.62–1.03)
	NO_x_, μg/m^3^	18.63 (12.37–26.00)
Average during the first year of life^a^	PM_2.5_, μg/m^3^	8.89 (8.37–9.57)
	PM_10_, μg/m^3^	14.65 (13.36–16.38)
	BC, μg/m^3^	1.03 (0.76–1.36)
	NO_x_, μg/m^3^	31.11 (20.38–44.05)

Abbreviations: BC, black carbon; NO_2_, nitrogen dioxide; NO_x_, nitrogen oxides; PM_2.5_, particulate matter with diameter ≤2.5 μm; PM_10_, particulate matter with diameter ≤10 μm.

a Calendar years of 16-y to 24-y covering 2010–2019. Calendar years of 1-y to 16-y covering 1994–2013. Calendar years of first year of life covering 1994–1996.

Table 3 shows the association between air pollution exposure and long COVID from the single pollutant models. We observed significant positive associations between PM_2.5_ exposure in 2019 and having long COVID in model 1 (OR = 1.25, 95% CI: 1.00–1.55 per IQR increase in PM_2.5_ exposure), which persisted in fully adjusted model 2 (OR = 1.28, 95% CI: 1.02–1.60 per IQR increase). Positive albeit non-significant associations were also found for other air pollution exposures in 2019 in Model 2: PM_10_ (OR = 1.20, 95% CI: 0.98–1.46), BC (OR = 1.13, 95% CI: 0.91–1.39), NO_x_ (OR = 1.14, 95% CI: 0.94–1.38). Similar associations were found using the 16-y to 24-y time-weighted average exposure, while no associations were found between air pollution exposure in the first year of life or 1-y to 16-y follow-up and having long COVID. Exposure-response curves showed the associations between air pollution and long COVID tend to be linear with no evidence of a threshold (Fig. S4).

**Table 3:** Adjusted odds ratios (ORs) and 95% confidence intervals (CIs) between air pollution exposure and long COVID.

Exposure window	Air pollutant	OR (95%CI)	
		Model 1^a^	Model 2^a^
2019 Annual average	PM_2.5_	1.25 (1.00, 1.55)	1.28 (1.02, 1.60)
	PM_10_	1.18 (0.97, 1.42)	1.20 (0.98, 1.46)
	BC	1.13 (0.92, 1.38)	1.13 (0.91, 1.39)
	NO_x_	1.15 (0.94, 1.38)	1.14 (0.94, 1.38)
16-y to 24-y time weighted average^b^	PM_2.5_	1.11 (0.84, 1.44)	1.28 (0.94, 1.72)
	PM_10_	1.09 (0.85, 1.36)	1.17 (0.90, 1.50)
	BC	1.02 (0.77, 1.32)	1.13 (0.82, 1.52)
	NO_x_	1.01 (0.79, 1.27)	1.08 (0.82, 1.40)
1-y to 16-y time weighted average^a^	PM_2.5_	0.92 (0.70, 1.22)	0.95 (0.67, 1.34)
	PM_10_	0.97 (0.73, 1.27)	1.00 (0.71, 1.39)
	BC	0.96 (0.71, 1.27)	0.98 (0.68, 1.40)
	NO_x_	0.96 (0.72, 1.27)	0.98 (0.68, 1.39)
Average during the first year of life^b^	PM_2.5_	0.98 (0.75, 1.26)	1.02 (0.69, 1.47)
	PM_10_	1.00 (0.77, 1.27)	1.05 (0.73, 1.45)
	BC	1.02 (0.76, 1.34)	1.10 (0.72, 1.62)
	NO_x_	1.02 (0.76, 1.34)	1.11 (0.73, 1.63)

Abbreviations: BC, black carbon; NO_x_, nitrogen oxides; PM_2.5_, particulate matter with diameter ≤2.5 μm; PM_10_, particulate matter with diameter ≤10 μm.

a Model 1 adjusted for age, sex and municipality at birth. Model 2 adjusted for age, sex, municipality at birth, education at 24-year, occupation at 24-year, smoking at 24-year, overweight at 24-year, physical activity at 24-year. ORs (95% CI) were presented per interquartile range increase.

b Calendar years of 16-y to 24-y follow-ups covering 2010–2019. Calendar years of 1-y to 16-y covering 1994–2013. Calendar years of the first year of life covering 1994–1996.

In subgroup analysis (Table S3), we observed that the effect estimates of air pollution exposure on long COVID were similar between males and females, normal weight and overweight, while stronger associations were observed among participants who ever had asthma (for PM_2.5_, OR = 1.39, 95% CI: 0.96–2.02) compared with those who never had asthma (OR = 1.16, 95% CI: 0.83–1.62), and those having the acute infection in 2020 (for PM_2.5_, OR = 1.32, 95% CI: 1.05–1.67) compared with 2021 (OR = 1.19, 95% CI: 0.94–1.51), but all the differences were non-significant (p values for interaction > 0.05).

**Fig. 1:**
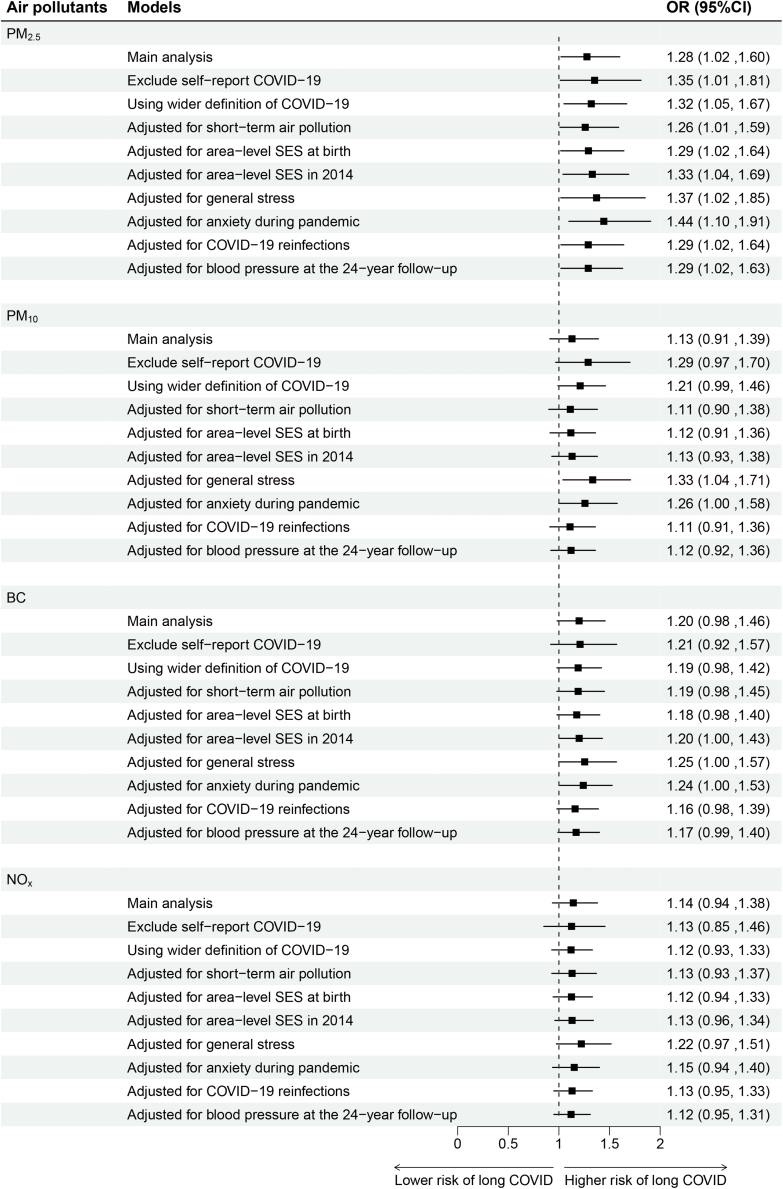
Sensitivity analyses of the association between air pollution exposure and long COVID. Results were adjusted for age, sex, municipality at birth, education at 24-year, occupation at 24-year, smoking at 24-year, overweight at 24-year, physical activity at 24-year. Abbreviations: BC, black carbon; NO_x_, nitrogen oxides; PM_2.5_, particulate matter with diameter ≤2.5 μm; PM_10_, particulate matter with diameter ≤10 μm. ORs (95%CI) were presented per interquartile range increase.

The observed associations between long-term air pollution exposure and having long COVID were consistent across the sensitivity analyses including restricted to participants with objective test results of COVID, additionally adjusted for short-term air pollution, additionally adjusted for area-level SES both at baseline or during the follow-up, additionally adjusted for general stress before the pandemic, anxiety during the pandemic, additionally adjusted for COVID-19 reinfections, using a wider COVID-19 definition (resulting in N = 1076 study subjects) or additionally adjusted for blood pressure at the 24-year follow-up, with even higher, significant point estimates in the additionally adjusted models (Fig. 1). Separate analysis on specific symptoms showed that air pollution was significantly associated with shortness of breath (for PM_2.5_, OR = 1.65, 95% CI: 1.09–2.50). Positive associations were also suggested for altered smell or taste (for PM_2.5_, OR = 1.29, 95% CI: 0.97–1.70), while no associations were observed for fatigue (Table S4). Only PM_2.5_ exposure remained statistically significant (OR = 1.42 95% CI: 1.01–2.00) in the multi-pollutant model (Table S5).

## Discussion

To our knowledge this is the first report of individual-level long-term exposure to air pollution associated with long COVID among young adults. In this population-based prospective cohort of 753 participants with SARS-CoV-2 infection, we found pre-pandemic long-term exposure to PM_2.5_ was significantly associated with increased risk of having long COVID. For each IQR increase in PM_2.5_ exposure in 2019, the odds of having long COVID increased by approximately 30%. No significant interactions were found by sex, overweight, asthma, allergic sensitization, prior respiratory disorders, COVID-19 severity and calendar year, indicating a general association of long-term air pollution exposure with post COVID-19 conditions.

Previously only one study reported positive association between PM_2.5_ exposure and persistent dyspnea after hospitalization due to COVID-19 among 749 adult patients in Brazil.^29^ Our results are in line with the previous findings, and further add to the evidence that outdoor air pollution could impact not only patients with severe COVID, but also young adults with mild or moderate symptoms on the odds of post COVID-19 conditions. We assessed the associations of four air pollutants with long COVID, with the strongest associations observed for PM_2.5_ while positive but not significant associations were observed for other pollutants. We also assessed different air pollution exposure time windows and found more pronounced associations with 2019 annual average or 16-y to 24-y follow-up average (calendar years from 2010 to 2019) than associations with the exposure in the first year of life, suggesting that more recent exposure could be more relevant for the prevalence of post COVID-19 conditions in our study.

We observed stronger association of PM_2.5_ exposure with long COVID than those for PM_10_, BC and NO_x_. The local emission sources for these pollutants mainly included road traffic (exhaust and non-exhaust), residential heating (defined as emissions from stoves and boilers in single-family houses as well as stoves used in apartments and small boilers used for heating multi-family houses), shipping and others (industrial processes, energy production, off-road machinery, agriculture, etc).^42^ By comparing the spatial distribution of PM_2.5_ and NO_x_ from traffic and residential heating sources (Fig. S5), we observed relatively high PM_2.5_ exposure but not NO_x_ exposure in areas with abundant residential heating (mainly from combustion of solid fuels), suggesting that residential heating plays a role in the stronger association observed for PM_2.5_ with long COVID than for other pollutants.

Among the symptoms of long COVID, strongest associations were found for shortness of breath with air pollution, which is consistent with the previous findings in Brazil.^29^ Positive associations were also found between PM_2.5_ exposure and altered sense of smell/taste. Interestingly, PM_2.5_ exposure has been reported to be associated with significantly faster decline of odor identification in a population-based sample of older adults in the Stockholm region, Sweden.^43^ These findings together indicate potential health impact of air pollution on brain health apart from the established adverse effect on cardiovascular and respiratory outcomes.^28^

Although it is difficult to infer causal mechanisms from an observational study, a reasonable explanation for our association (based on prior knowledge about air pollution mechanisms) is that air pollution exposure triggers inflammation/hyper-sensitivity in vulnerable subjects also affected by COVID-19. Thus, a combination of susceptibility factors (including genetic, epigenetic, immunologic factors), previous and current air pollution exposure with SARS-Cov2 infection is likely contributing to long COVID. Ambient air pollution exposure have been shown to be positively associated with increased level of inflammatory cytokines^38^ and proteins,^44^, ^45^, ^46^ which have been proposed as possible causes of multiple symptoms of post COVID-19 conditions.^3^ In addition, air pollution has been found to be associated with immune dysregulation including immune suppression,^24^^,^^47^ which was reported to be associated with risk of persistent symptoms after COVID-19.^48^ The virulence of the SARS-CoV-2 virus may be alter in polluted region as a contributing mechanism.^49^ Furthermore, long-term air pollution exposure can have adverse effect on a wide range of comorbidities^28^ such as cardiometabolic diseases and respiratory diseases, which has been found to be associated with risk of developing long COVID.^50^ This is consistent with our results that the observed association tended to be stronger among participants with asthma. However, we were unable to investigate the potential role of cardiometabolic diseases (beyond blood pressure) due to the relatively young ages of our study population.

Strengths of the current study includes well-characterized study population, individual level air pollution exposure estimated by spatiotemporal model with high resolution in different time-windows and the rich information on covariates and potential effect modifiers. However, our results should be interpreted bearing in mind some limitations. First, only association not causality can be concluded due to the observational study design. Second, outcome misclassification for those participants who self-reported as having COVID may be of concern, but in the sensitivity analysis restricted to participants with objective confirmation of COVID (Fig. 1), we still observed positive significant associations between air pollution and long COVID. In addition, applying an even wider definition of long COVID (including self-report of *suspected* COVID, totaling N = 1076 study subjects) also gave significant results. We did not investigate the occurrence of symptoms defining long COVID among individuals who never had COVID and it cannot be ruled out that some of the symptoms may also have association with air pollution in that group. Third, residual confounding cannot be completely ruled out. We performed sensitivity analyses additionally adjusting for factors which could be potentially associated with both long-term air pollution exposure and long COVID, including short-term air pollution, socioeconomic status, stress and anxiety,^51^ and associations between long-term air pollution and long COVID were in general robust (Fig. 1). Fourth, potential exposure misclassification may also exist due to the lack of exposure during the pandemic period (2020–2022) as well as the modeled ambient air pollution exposure. While true personal exposure to air pollutants might be seen as ideal in a purely etiological context, outdoor concentrations have the advantages of not introducing confounding due to personal habits and of being more accessible for monitoring and regulation. In addition, our estimates of outdoor levels were modelled in a fine spatial gridnet, minimizing problems of systematic spatial misalignment between exposure and outcome. Fifth, only 26 participants had been vaccinated before infected with SARS-CoV-2, which made us underpowered to investigate the association between air pollution and long COVID among the vaccinated young adults. Relatively small sample size also limited our statistical power to identify possible interactions across different subgroups. Future studies with large sample size and information on pre-pandemic covariates are needed to investigate potential effect modifiers. In addition, the different characteristics between respondents and non-respondents may influence the generalizability of our results.

In conclusion, the findings in this cohort study of Swedish young adults suggest that residential long-term exposure to air pollution was associated with increased risk of having post COVID-19 conditions after SARS-CoV-2 infection. These findings shed light on the complex pathogenesis of long-term post COVID-19 symptoms and ubiquitous adverse health effects of air pollutants. Since ambient air pollution is modifiable risk factor through national or regional public health regulations as well as individual interventions, our results support the broad public health benefits of continuous efforts to reduce ambient air pollution levels.

## Contributors

Z.Y., O.G., E.M., G.P., T.B., P.L. contributed to the conception and design of the study. Z.Y., O.G., E.M. had full access to all the data in the study. Z.Y. did the statistical analysis and wrote the initial draft under the supervision of O.G. and E.M. E.M. had final responsibility for the decision to submit for publication. E.M., A.B., A.G., I.K. acquired the data. K.E., O.G., T.B., G.P. contributed to the assessment of air pollution exposure. O.G., E.M., A.B., A.G., I.K. obtained funding for the study. S.E., M.S. contributed to the interpretation of the data. All authors contributed to the revision the manuscript for important intellectual content, approved for the final version, and agreed to be accountable for all aspects of the work.

## Data sharing statement

The datasets analyzed during the current study are not publicly available but the derived data supporting the findings of this study are available from the corresponding author (EM) on reasonable request.

## Declaration of interests

The authors have nothing to disclose.

## Appendix A Supplementary data

Tables S1–S5 and Figures S1–S4

Translated Abstract
