# Supplementary material for: Ambient air pollution exposure linked to long COVID among young adults: a nested survey in a population-based cohort in Sweden
Source: Lancet Reg Health Eur. 2023 Mar 7;28:100608. doi: 10.1016/j.lanepe.2023.100608 (PMC9989696; doi:10.1016/j.lanepe.2023.100608)
Supplement: Tables S1–S5 and Figures S1–S4 [file mmc1.docx]

**Ambient air pollution exposure linked to long COVID among young adults: a nested survey in a population-based cohort in Sweden**

**Online supplemental material**

**eMethods** Air pollution exposure assessment

**Table E1** Characteristics of the participants who responded and did not responded to the COVID phase-3 questionnaire follow-up

**Table E2** Distribution of the air pollution exposure in different exposure time windows

**Table E3** Subgroup analysis of the association between air pollution and long COVID

**Table E4** Adjusted association between air pollution and specific post COVID-19 symptom cluster and single symptoms

**Table E5** Adjusted OR (95%CI) of air pollution exposure with long covid in single exposure model and multi-exposure models using ridge penalty

**Figure E1**. Flowchart of inclusion of the participants in the current study

**Figure E2**. Number of participants infection with SARS-CoV-2 identified from different sources.

**Figure E3**. Spearman correlations between long-term air pollution exposure in different exposure windows.

**Figure E4**. Estimated exposure-response curves of air pollution exposure on long COVID.

**eMethods**

**Air pollution exposure assessment**

Road traffic emissions of NO_x_ and PM-exhaust for different vehicle types, speeds and driving conditions were calculated according to the European emission model HBEFA (<https://www.hbefa.net/e/index.html>). Emission factors for BC were estimated based on the fraction of BC in PM-exhaust. This fraction varies depending on fuel and type of vehicle and the information was derived from the Transphorm project (<http://www.transphorm.eu/>). Emission factors for non-exhaust PM, including road wear and some contributions from brake and tire wear, were obtained from Omstedt et al. (2005)^2^ and from the NORTRIP model^3,4^. In Stockholm during the late winter and in connection with dry road surfaces, the contribution from studded tire wear can be 80 % to 90 % of the total PM_10_ levels. The road wear PM also makes a considerable contribution to the total PM_2.5_ levels.

To obtain total air pollution concentrations, annual average long-range contributions based on continuous measurements at regional background stations, were added to the locally modelled concentrations. Due to low availability of monitored BC (the measurements started in 2007), and observed low year-to-year variability in existing measurement data of the related metric black smoke, the long-range transport contributions of BC were assumed constant over the period 1990 – 2012, i.e. 0.3 µg/m^3^. For NO_x_, measurement data were available from 1994. The long-distance contribution for previous years has been estimated on the basis of extrapolation of measurement data back in time. For PM_10_ and PM_2.5_, long-distance contributions from 2006 onwards consist of measured concentrations at a background monitoring station approx. 50 km northeast of Stockholm. For the years before 2006, the long-distance contributions of PM_10_ and PM_2.5_ have been estimated by combining measurement data from two regional background stations and its relationship to measured concentrations at an urban background station in central Stockholm.

**Reference**

1. Gruzieva O, Merid SK, Gref A, et al. Exposure to Traffic-Related Air Pollution and Serum Inflammatory Cytokines in Children. *Environ Health Perspect* 2017; **125**(6): 067007.

2. Omstedt G, Bringfelt B, Johansson C. A model for vehicle-induced non-tailpipe emissions of particles along Swedish roads. *Atmospheric environment* 2005; **39**(33): 6088-97.

3. Denby BR, Sundvor I, Johansson C, et al. A coupled road dust and surface moisture model to predict non-exhaust road traffic induced particle emissions (NORTRIP). Part 1: Road dust loading and suspension modelling. *Atmospheric Environment* 2013; **77**: 283-300.

4. Denby BR, Sundvor I, Johansson C, et al. A coupled road dust and surface moisture model to predict non-exhaust road traffic induced particle emissions (NORTRIP). Part 2: Surface moisture and salt impact modelling. *Atmospheric Environment* 2013; **81**: 485-503.

**Table E1** **Characteristics of the participants who responded and did not responded to the COVID phase-3 questionnaire follow-up**

| Characteristics^*^ | Respondent (n=2049) | Non-respondent (n=932) | P values |
| --- | --- | --- | --- |
| Sex |  |  | <0.001 |
| Male | 840 (41.0%) | 552 (59.2%) | - |
| Female | 1209 (59.0%) | 380 (40.8%) | - |
| Age, years | 22.4±0.5 | 22.6±0.6 | <0.001 |
| BMI | 23.0±3.8 | 23.5±4.0 | 0.004 |
| Education level |  |  | 0.003 |
| Elementary school or high school | 775 (37.8%) | 308 (33.0%) | - |
| University or college | 1274 (62.2%) | 624 (67.0%) | - |
| Occupation |  |  | <0.001 |
| Studying | 1138 (55.5%) | 394 (42.3%) | - |
| Employed | 769 (37.5%) | 441 (47.3%) | - |
| Other | 142 (6.9%) | 97 (10.4%) | - |
| Active smoking | 383 (18.7%) | 233 (25.0%) | <0.001 |
| Air pollution exposure in 2019 |  |  | - |
| PM_2.5_, μg/m^3^ | 6.4±0.6 | 6.3±0.6 | 0.204 |
| PM_10_, μg/m^3^ | 11.7±1.8 | 11.6±1.8 | 0.037 |
| BC, μg/m^3^ | 0.35±0.08 | 0.34±0.08 | 0.033 |
| NO_x_, μg/m^3^ | 12.3±8.0 | 11.6±7.9 | 0.012 |

All the characteristics were from the 24-year questionnaire follow-up. Results are presented as frequency (percentage) for categorical variables and mean±standard deviation for continuous variables.

**Table E2 Distribution of the air pollution exposure in different exposure time windows**

| Exposure time window | Air pollutants | P5 | P25 | P50 | P75 | P95 | Mean | SD | IQR |
| --- | --- | --- | --- | --- | --- | --- | --- | --- | --- |
| 2019 Annual average | PM_2.5_, μg/m^3^ | 5.42 | 6.06 | 6.39 | 6.71 | 7.37 | 6.38 | 0.59 | 0.65 |
|  | PM_10_, μg/m^3^ | 9.64 | 10.63 | 11.54 | 12.36 | 14.97 | 11.76 | 1.74 | 1.72 |
|  | BC, μg/m^3^ | 0.25 | 0.30 | 0.34 | 0.37 | 0.51 | 0.35 | 0.08 | 0.07 |
|  | NO_x_, μg/m^3^ | 5.19 | 7.39 | 10.47 | 14.27 | 30.07 | 12.76 | 8.15 | 6.88 |
|  | NO_2_, μg/m^3^ | 4.66 | 6.5 | 9.01 | 11.69 | 20.64 | 10.11 | 4.98 | 5.19 |
| 16-y to 24-y time weighted average^*^ | PM_2.5_, μg/m^3^ | 4.51 | 5.12 | 5.49 | 5.77 | 6.56 | 5.49 | 0.65 | 0.65 |
|  | PM_10_, μg/m^3^ | 10.73 | 11.8 | 12.78 | 13.6 | 16.68 | 13.06 | 2.02 | 1.79 |
|  | BC, μg/m^3^ | 0.31 | 0.38 | 0.47 | 0.55 | 0.83 | 0.5 | 0.17 | 0.16 |
|  | NO_x_, μg/m^3^ | 5.86 | 8.33 | 12.20 | 17.86 | 37.76 | 15.55 | 10.90 | 9.53 |
| 1-y to 16-y time weighted average^*^ | PM_2.5_, μg/m^3^ | 6.78 | 7.30 | 7.77 | 8.18 | 9.01 | 7.79 | 0.69 | 0.88 |
|  | PM_10_, μg/m^3^ | 11.13 | 11.99 | 13.22 | 14.32 | 16.56 | 13.42 | 1.81 | 2.33 |
|  | BC, μg/m^3^ | 0.51 | 0.62 | 0.80 | 1.03 | 1.42 | 0.86 | 0.30 | 0.41 |
|  | NO_x_, μg/m^3^ | 9.02 | 12.37 | 18.63 | 26.00 | 39.59 | 20.56 | 10.28 | 13.63 |
| Average during the first year of life | PM_2.5_, μg/m^3^ | 7.65 | 8.37 | 8.89 | 9.57 | 10.55 | 9.02 | 1.02 | 1.2 |
|  | PM_10_, μg/m^3^ | 11.93 | 13.36 | 14.65 | 16.38 | 18.7 | 15.09 | 2.67 | 3.02 |
|  | BC, μg/m^3^ | 0.55 | 0.76 | 1.03 | 1.36 | 1.81 | 1.1 | 0.47 | 0.6 |
|  | NO_x_, μg/m^3^ | 12.65 | 20.38 | 31.11 | 44.05 | 60.83 | 34.03 | 18.82 | 23.67 |

P5, 5^th^ percentile; P25, 25^th^ percentile; P50, 50^th^ percentile; P75, 75^th^ percentile; P95, 95^th^ percentile; SD, standard deviation; IQR, interquartile range

PM_2.5_, particulate matter with diameter ≤ 2.5 μm; PM_10_, particulate matter with diameter ≤ 10 μm; BC, black carbon; NO2, nitrogen dioxides; NOx, nitrogen oxides.

*: calendar years of 16-y to 24-y follow-ups covering 2010 to 2019. Calendar years of 1-y to 16-y follow-ups covering 1994 to 2013. Calendar years of first year of life covering 1994 to 1996.

**Table E3** Subgroup analysis of the association between air pollution and long COVID

| Category/air pollutant | Subgroups | | P-value for interaction |
| --- | --- | --- | --- |
|  | OR (95%CI)^*^ | OR (95%CI)^*^ |  |
| Sex | Female | Male | - |
| PM_2.5_ | 1.28 (1.03 ,1.61) | 1.26 (1.00 ,1.58) | 0.278 |
| PM_10_ | 1.14 (0.92 ,1.41) | 1.10 (0.88 ,1.37) | 0.271 |
| BC | 1.24 (1.02 ,1.52) | 1.14 (0.92 ,1.41) | 0.090 |
| NO_x_ | 1.22 (0.99 ,1.50) | 1.01 (0.79 ,1.30) | 0.076 |
| BMI category | Normal weight | Overweight | - |
| PM_2.5_ | 1.27 (1.02 ,1.59) | 1.28 (1.02 ,1.61) | 0.799 |
| PM_10_ | 1.13 (0.91 ,1.40) | 1.13 (0.90 ,1.41) | 0.847 |
| BC | 1.19 (0.98 ,1.45) | 1.19 (0.96 ,1.48) | 0.887 |
| NO_x_ | 1.16 (0.94 ,1.42) | 1.10 (0.82 ,1.47) | 0.548 |
| Asthma status | No asthma | Asthma | - |
| PM_2.5_ | 1.16 (0.83 ,1.62) | 1.39 (0.96 ,2.02) | 0.289 |
| PM_10_ | 1.06 (0.77 ,1.47) | 1.33 (0.96 ,1.86) | 0.259 |
| BC | 1.05 (0.78 ,1.41) | 1.40 (0.99 ,1.97) | 0.199 |
| NO_x_ | 1.17 (0.89 ,1.55) | 1.25 (0.89 ,1.75) | 0.668 |
| Allergic sensitization | Negative | Positive | - |
| PM_2.5_ | 1.24 (0.90 ,1.72) | 1.36 (0.90 ,2.04) | 0.694 |
| PM_10_ | 1.20 (0.86 ,1.66) | 1.27 (0.90 ,1.79) | 0.725 |
| BC | 1.18 (0.87 ,1.60) | 1.31 (0.95 ,1.81) | 0.852 |
| NO_x_ | 1.16 (0.87 ,1.55) | 1.09 (0.79 ,1.52) | 0.374 |
| Respiratory disorders at 24-y | No | Yes |  |
| PM_2.5_ | 1.27 (1.01 ,1.59) | 1.31 (1.05 ,1.65) | 0.649 |
| PM_10_ | 1.10 (0.89 ,1.37) | 1.17 (0.95 ,1.46) | 0.674 |
| BC | 1.15 (0.90 ,1.46) | 1.30 (0.89 ,1.89) | 0.466 |
| NO_x_ | 1.07 (0.87 ,1.33) | 1.31 (1.03 ,1.66) | 0.522 |
| COVID-19 severity | No bedridden | Bedridden | - |
| PM_2.5_ | 1.21 (0.96 ,1.52) | 1.26 (1.01 ,1.58) | 0.972 |
| PM_10_ | 1.07 (0.86 ,1.33) | 1.15 (0.93 ,1.42) | 0.836 |
| BC | 1.10 (0.88 ,1.36) | 1.21 (0.99 ,1.48) | 0.816 |
| NO_x_ | 0.97 (0.74 ,1.27) | 1.21 (0.99 ,1.49) | 0.780 |
| Calendar year | 2020 | 2021 | - |
| PM_2.5_ | 1.32 (1.05 ,1.67) | 1.19 (0.94 ,1.51) | 0.239 |
| PM_10_ | 1.16 (0.93 ,1.43) | 0.99 (0.78 ,1.24) | 0.395 |
| BC | 1.15 (0.91 ,1.46) | 0.94 (0.72 ,1.23) | 0.154 |
| NO_x_ | 1.19 (0.97 ,1.46) | 0.96 (0.75 ,1.26) | 0.144 |

PM_2.5_, particulate matter with diameter ≤ 2.5 μm; PM_10_, particulate matter with diameter ≤ 10 μm; BC, black carbon; NO_x_, nitrogen oxides. Annual average air pollution exposure in 2019 was used as the exposure.

*: Results were adjusted for age, sex, municipality at birth, education at 24-year, occupation at 24-year, smoking at 24-year, overweight at 24-year, physical activity at 24-year.

**Table E4** Adjusted association between air pollution and specific post COVID-19 symptom cluster and single symptoms

|  |  | Number of participants with/without symptoms | ORs (95%CI) | | | |
| --- | --- | --- | --- | --- | --- | --- |
| **Symptom clusters** | **Specific symptoms within cluster** |  | PM_2.5_ | PM_10_ | BC | NO_x_ |
|  |  |  |  |  |  |  |
| General symptoms | Fatigue, muscle weakness, pain | 44/637 | 1.08 (0.74, 1.58) | 0.93 (0.66, 1.31) | 0.92 (0.68, 1.25) | 0.96 (0.72, 1.29) |
| Respiratory or heart problems | Shortness of breath, High resting heart rate or palpitations | 43/637 | 1.58 (1.08, 2.29) | 1.31 (0.97, 1.76) | 1.38 (1.07, 1.78) | 1.33 (1.05, 1.68) |
| Neurological symptoms | Altered sense of smell and taste, headache, cognitive impairment, neurological symptoms, mental illness, sleep disorders | 102/637 | 1.25 (0.97, 1.60) | 1.03 (0.83, 1.29) | 1.12 (0.93, 1.36) | 1.04 (0.86, 1.26) |
| **Single symptoms** |  |  |  |  |  |  |
| Altered smell and taste |  | 80/637 | 1.29 (0.97 ,1.70) | 1.12 (0.88 ,1.43) | 1.16 (0.93 ,1.44) | 1.10 (0.88 ,1.35) |
| Fatigue |  | 34/637 | 0.83 (0.50 ,1.43) | 0.83 (0.49 ,1.36) | 0.80 (0.49 ,1.24) | 0.82 (0.49 ,1.25) |
| Shortness of breath |  | 36/637 | 1.65 (1.09 ,2.50) | 1.39 (1.00 ,1.91) | 1.46 (1.09 ,1.91) | 1.33 (1.01 ,1.72) |

Abbreviations: PM_2.5_, particulate matter with diameter ≤ 2.5 μm; PM_10_, particulate matter with diameter ≤ 10 μm; BC, black carbon; NO_x_, nitrogen oxides.

Air pollution exposure of 2019 annual average was used. All results were adjusted for age, sex, municipality at birth, education at 24-year, occupation at 24-year, smoking at 24-year, overweight at 24-year, physical activity at 24-year.

**Table E5** Adjusted OR (95%CI) of air pollution exposure with long COVID in single exposure model and multi-exposure models using ridge penalty

| Air pollutants | Single exposure model | Multi-exposure model using ridge penalty |
| --- | --- | --- |
| PM_2.5_ | 1.28 (1.02, 1.60) | 1.42 (1.01, 2.00) |
| PM_10_ | 1.20 (0.98, 1.46) | 1.29 (0.77, 2.16) |
| BC | 1.13 (0.91, 1.39) | 1.49 (0.89, 2.50) |
| NOx | 1.14 (0.94, 1.38) | 1.38 (0.81, 2.34) |

Results were adjusted for age, sex, municipality at birth, education at 24-year, occupation at 24-year, smoking at 24-year, overweight at 24-year, physical activity at 24-year. ORs (95%CI) were presented as per interquartile range increase. Air pollution exposure in 2019 was used.


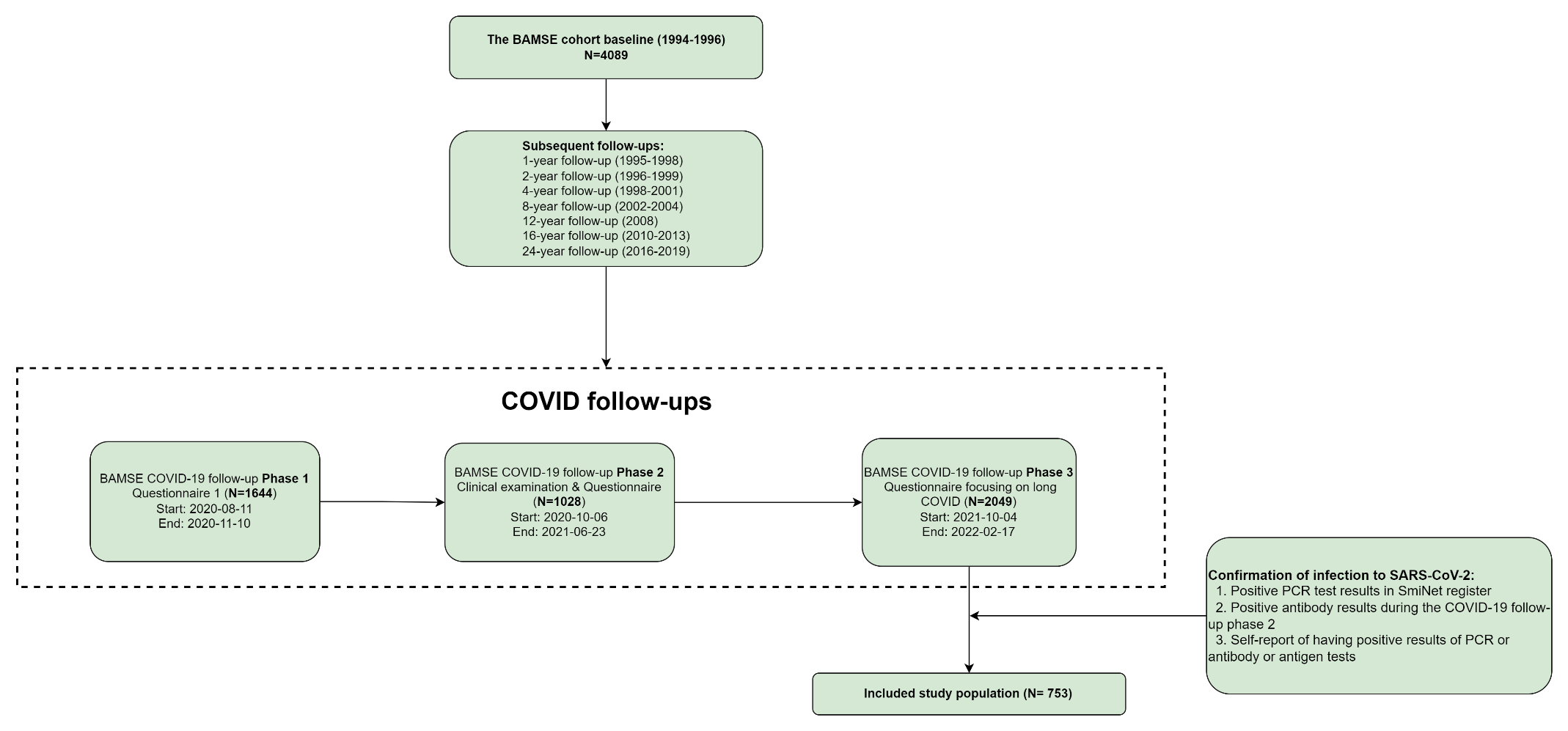


**Figure E1**. Flowchart of inclusion of the participants in the current study


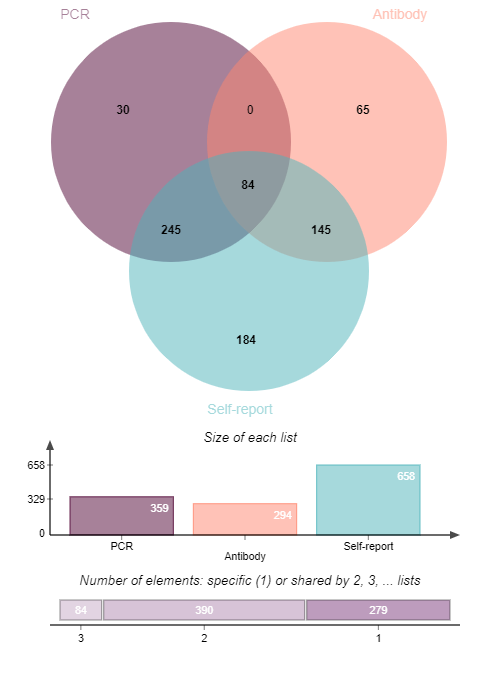


**Figure E2**. Number of participants infection with SARS-CoV-2 identified from different sources.


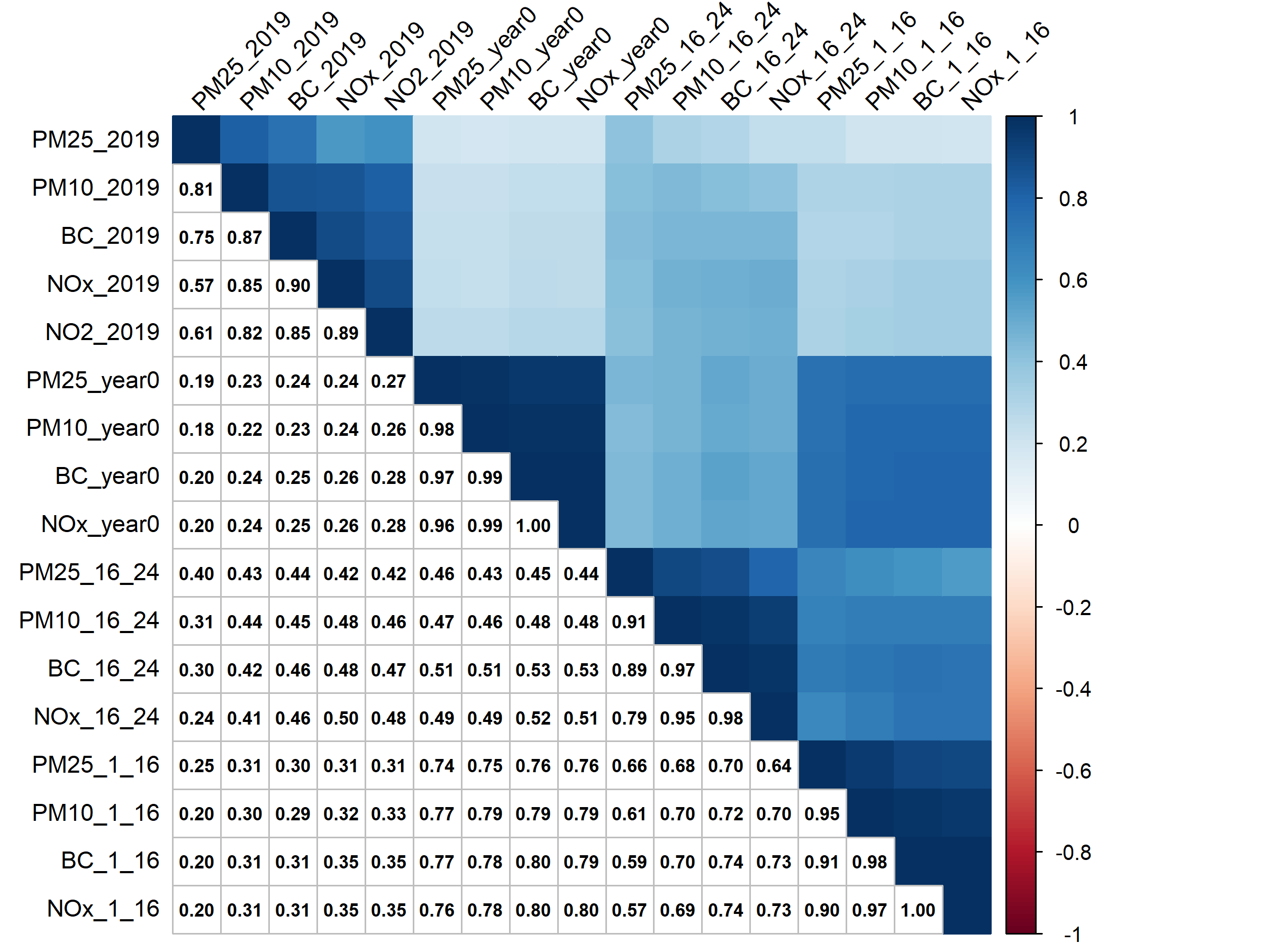


**Figure E3**. Spearman correlations between long-term air pollution exposure in different exposure windows.


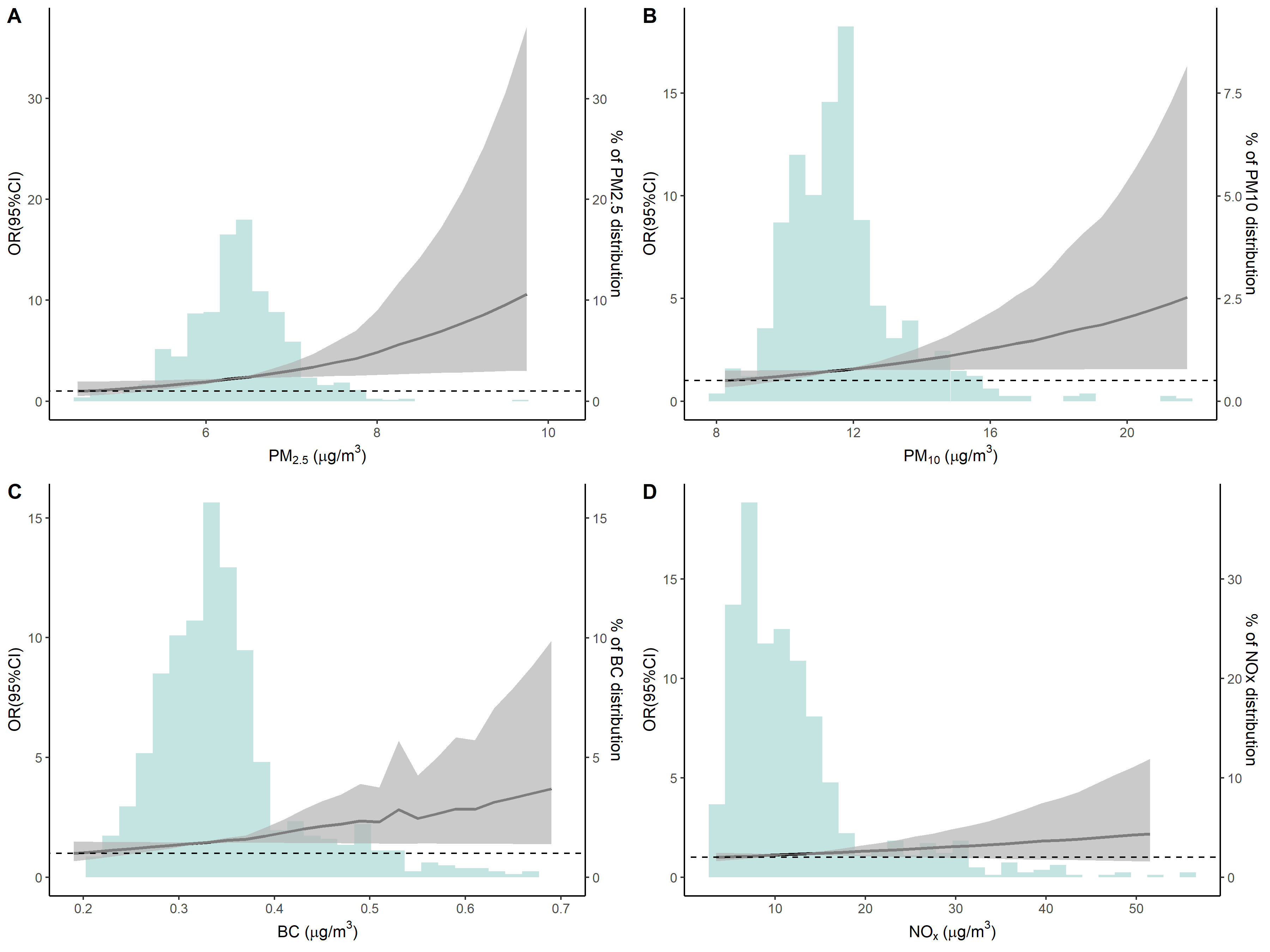


**Figure E4** Estimated exposure-response curves of air pollution exposure on long COVID. A) particulate matter with diameter ≤ 2.5 μm; B) particulate matter with diameter ≤ 10 μm; C) black carbon; D) nitrogen oxides. Natural cubic splines with three degrees of freedom were fit based on the Model 2, adjusting for age, sex, municipality at birth, education at 24-year, occupation at 24-year, smoking at 24-year, overweight at 24-year, physical activity at 24-year. Black solid line indicated odds ratio, and grey area indicated 95% confidence interval. The background green histogram shows the distribution of air pollution among participants.


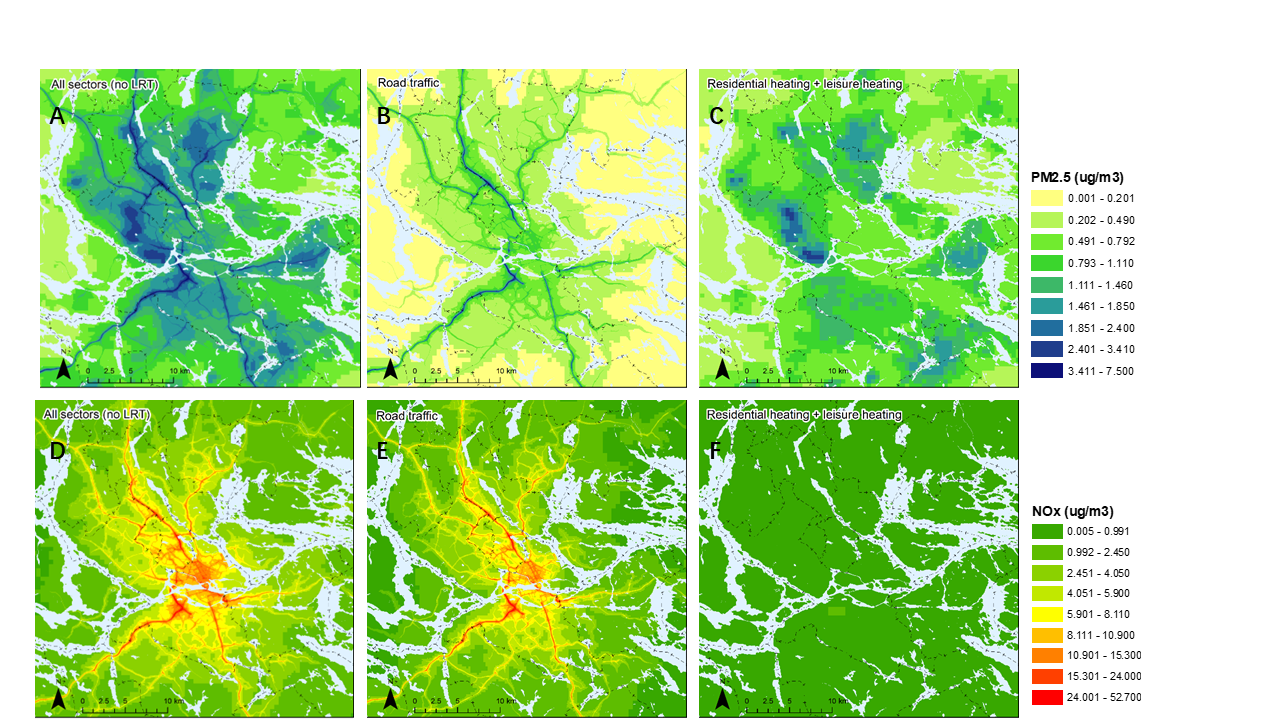


**Figure E5** Spatial distribution of PM_2.5_ and NOx in the study region. (A) PM_2.5_ distribution from all emission sources; (B) PM_2.5_ distribution from road traffic: (C) PM_2.5_ distribution from residential heating; (D) NOx distribution from all emission sources; (E) NOx distribution from road traffic; (F) NOx distribution from residential heating. Coordinates of participants were not presented to avoid potential identification. Distribution of air pollution in 2020 is presented, with the same geographical distribution as distribution in 2019.
