# Supplementary material for: Ambient air pollution exposure linked to long COVID among young adults: a nested survey in a population-based cohort in Sweden
Source: Lancet Reg Health Eur. 2023 Mar 7;28:100608. doi: 10.1016/j.lanepe.2023.100608 (PMC9989696; doi:10.1016/j.lanepe.2023.100608)
Supplement: Translated Abstract [file mmc2.docx]

**Translated abstract, Yu et al**

**Disclaimer: *The following translation in Swedish was submitted by the authors and we reproduce them as supplied. They have not been peer reviewed. Our editorial processes have only been applied to the original abstract in English, which should serve as reference for this manuscript.***

**Samband mellan exponering för luftföroreningar och postcovid bland unga vuxna: resultat från en populationsbaserad kohort i Sverige**

**Bakgrund** Postcovid, eller ”long covid”, utgör idag ett stort folkhälsoproblem, men lite är känt om underliggande riskfaktorer. Vårt syfte var att undersöka samband mellan luftföroreningar och långvariga covidsymptom hos unga vuxna i Sverige.

**Metoder** Vi använde data från kohorten BAMSE (Barn, Allergi, Miljö, Stockholm, Epidemiologi). Från oktober 2021 till februari 2022 besvarade deltagarna ett webbfrågeformulär med fokus på långvariga symtom efter SARS-CoV-2-infektion. Postcovid definierades som symtom som varat i två månader eller längre efter bekräftad infektion med SARS-CoV-2. Luftföroreningsnivåerna (partiklar≤2.5 μm [PM_2.5_], ≤10 μm [PM_10_], sot [BC] och kväveoxider [NO_x_]) på deltagarnas bostadsadresser uppskattades med hjälp av dispersionsmodellering.

**Resultat** Totalt 753 deltagare med SARS-CoV-2-infektion inkluderades i studien, varav 116 (15.4%) uppfyllde kriterier för postcovid. De vanligaste symtomen var förändrad lukt/smak (n=80, 10,6 %), dyspné (n=36, 4,8 %) och trötthet (n=34, 4,5 %). Medianvärdet för den årliga PM_2.5_-exponeringen 2019 (före pandemin) var 6.39 (interkvartilintervall [IQR] 6.06–6.71) μg/m^3^. Justerade oddskvoter (95% konfidensintervall) för PM_2.5_ per IQR-ökning var 1.28 (1.02-1.60) för postcovid, 1.65 (1.09-2.50) för andnöd och 1.29 (0.97-1.70) för förändrad lukt/smak. Positiva samband hittades även för de andra luftföroreningarna och i olika sensitivitetsanalyser. Sambanden tenderade att vara starkare bland deltagare med astma och de som hade haft covid under 2020 (jämfört med 2021).

**Tolkning** Långvarig PM_2.5_-exponering kan påverka risken för postcovid hos unga vuxna, vilket stöder insatser för att kontinuerligt förbättra luftkvaliteten.

**Finansiering** Studien finansierades av Vetenskapsrådet (nr. 2020-01886, 2022-06340), Forskningsrådet för hälsa, arbetsliv och välfärd (FORTE-anslag nr 2017-01146), Hjärt-Lungfonden, Karolinska Institutet (nr. 2022-01807) och Region Stockholm (ALF-projekt för kohort- och databasunderhåll).
